# Path analysis of strength, spasticity, gross motor function, and health-related quality of life in children with spastic cerebral palsy

**DOI:** 10.1186/s12955-018-0891-1

**Published:** 2018-04-19

**Authors:** Eun-Young Park

**Affiliations:** Department of Secondary Special Education, College of Education, Jeonju University, 1200 3-ga, Hyoja-dong, Wansan-gu, Jeonju, 560-759 South Korea

**Keywords:** Cerebral palsy, Health-related quality of life (QOL), Path analysis

## Abstract

**Background:**

Measures of health-related quality of life may predict the future status of individuals with illnesses, and could therefore be a good indicator in children with cerebral palsy (CP). This study examines the causal relationship between spasticity, weakness, gross motor function, and health-related quality of life (QOL) in school-aged children with spastic CP and tests models of functional outcome mediated by gross motor function.

**Methods:**

A total of 62 children (44 males, 18 females) with spastic CP were recruited. Strength was assessed with the Manual Muscle Test, spasticity with the Modified Ashworth Scale, and the Gross Motor Function Measure was also employed. Health-related QOL was assessed using the Korean version of the Childhood Health Assessment Questionnaire. Physical therapists interviewed the parents and assessed the children.

**Results:**

The proposed path model showed good fit indices. The direct effects were significant between spasticity and gross motor function, strength and gross motor function, gross motor function and health-related QOL, and strength and health-related quality of life. Spasticity had a significant positive indirect effect and strength a significant negative indirect effect on health-related QOL through gross motor function.

**Conclusion:**

This is an initial study of the causal relationship between strength, spasticity, gross motor function, and health-related QOL.

## Background

Cerebral palsy (CP) is defined as a permanent non-progressive developmental disorder. National population-based studies have reported that CP prevalence is about 1.8-2.3 cases per 1000 children in Europe, Australia, and the U.S. [1–3]. A fourth Canadian study reported that in Northern Alberta, the CP rate is 2.22 per 1000 5-year-old children [4]. Although children with CP receive medical treatment and rehabilitation, several limitations can still affect their functioning and abilities, including the ability to perform activities of daily living [5]. The range of developmental problems and comorbidities of children with CP, such as movement, sensory, perceptual, cognitive, communication, and behavior disorders, epilepsy, and secondary musculoskeletal problems [6], is varied and affects not only children’s functioning but also their quality of life (QOL) and family [7]. In the case of spastic CP, spasticity, especially when it is severe, has been recognized as a major factor in pain and mobility impairment, and its reduction has been emphasized as a treatment goal [8].

The International Classification of Functioning, Disability, and Health (ICF) model is concerned with the effect of disability on an individual’s daily life and social participation, beyond the idea of injury, which implies physical structure and functional deficiency. Since the conceptual model of ICF disability was presented, the reduction of disability and promotion of participation in individuals with disabilities has become the ultimate goal of rehabilitation and a health policy direction [9].

Recently, based on the ICF concepts, research on functional assessment measures (activity limitation and participation restriction) and QOL has been conducted to determine the direction and effect of interventions. The Gross Motor Function Measure (GMFM) is a tool for functional assessment, and its scores have a close relationship with motor impairments, including spasticity and decreased muscle strength. Kim and Park [10] reported a causal relationship between spasticity, muscle strength, and GMFM using path analysis. Furthermore, Ko et al. [11] reported that improvement of muscle strength in the lower extremities may have positive effects on social standards such as activity and participation among individuals with CP.

Severity of CP is associated with low physical QOL [12, 13]. QOL refers to the general well-being of individuals and societies, and is defined by the World Health Organization as “an individual’s perception of their position in life in the context of the culture and value systems in which they live, and in relation to their goals, expectations, standards and concerns” [14]. Since the concept of QOL is wide, it is difficult to measure. In children with CP with motor disturbances, mainly health-related QOL (HRQOL) is assessed, which refers to how individuals feel about aspects of their lives directly related to health, excluding issues such as religious beliefs and practices [15].

Previous studies have reported that gross motor function is related to QOL in children with CP. Gross motor function, measured by GMFM, and Gross Motor Function Classification System level were found to be highly correlated with physical well-being in school-aged children with CP [16]. Especially, GMFM scores were highly associated with QOL, and GMFM and mastery motivation explained 65% of the variance in the physical domain of QOL [16]. Not all studies have reported a relationship between motor impairment, gross motor function, and QOL [17, 18]. Arnaud et al. [19] suggested that, depending on the areas of life assessed, the most severely impaired children do not always have the poorest QOL. CP pain, which is common in high-tension children, is closely related to QOL [17, 20, 21]. Previous studies, however, have used univariate analysis, which is insufficient to confirm direct causal relationships.

Impairment-based therapy focuses on the inhibition of spasticity and improvement of muscle strength, while activity-based therapy focuses on the improvement of gross motor function. Given the recent emphasis on activity-based therapy in clinical practice, it is necessary to research the causal relationship between a variety of factors and how they affect HRQOL in spastic CP. The Childhood Health Assessment Questionnaire (CHAQ), a tool specifically developed to assess functional capacity and independence in everyday life, is a commonly used measure of health status. Although the CHAQ was designed to assess childhood idiopathic arthritis in children and adolescents [22], it has been shown to be useful for patients with other chronic disorders such as hypermobile children and juvenile spondyloarthropaty [23, 24], and has been applied to patients with current exercise limitations due to illness [25].

This study aimed to examine the causal relationship between spasticity, weakness, gross motor function, and HRQOL in children with spastic CP who attend school. Especially, the mediation effect of gross motor function was investigated.

## Methods

This study aimed to investigate the relationship between spasticity, weakness, gross motor function, and HRQOL in children with spastic CP who attend school using path analysis. This study employed a cross-sectional and prospective design. Informed consent was obtained from the parents of all children, as parents of children below 15 years of age are their legal representatives. Ethical approval for this study was granted by the Ethics Committee of Jeonju University.

Participants were 62 children with spastic CP, 44 (71.0%) boys and 18 (29.0%) girls. Inclusion criteria were as follows: 1) clinical diagnosis of spastic CP, 2) age from 6 to 15 years, and 3) consent of parents to participate. Exclusion criteria were as follows: 1) spina bifida, 2) associated skeletal muscular disorders such as muscular dystrophy and myopathy, and 3) elective dorsal rizotomy surgery. Botulinum toxin injection was not considered as inclusion/exclusion criterion. The mean age of children was 9.02 years (SD = 2.6). The minimum required sample size was 42 based on a significance level of 0.05 and medium effect size of 0.50 in a correlation analysis [26]. According to the Korean Gross Motor Function Classification System, 14 (22.6%), 14 (22.6%), 10 (16.1%), 7 (11.3%), and 17 (27.4%) children were classified under levels I–V, respectively. Regarding the type of spastic CP, quadriplegia was found in 16.1% of the participants, triplegia in 6.5%, diplegia in 51.6%, and hemiplegia in 25.8%. Children with spastic CP take part in physical and occupational therapy. Twelve children, 20% of the participants, received physical therapy once a week, 23 (36.4%) were treated twice a week, 13 (20.7%) were treated three times per week, and the remaining children received treatment four or more times per week. Fourteen children (23.1% of the participants), received occupational therapy once a week, 20 (32.4%) were treated twice a week, 20 (32.4%) received occupational therapy three times per week, and the remaining children received treatment four or more times per week. In this study, 70% of the children with spastic CP had experienced botulinum toxin injection.

A spasticity rating was obtained through bilateral measurement of spasticity using the Modified Ashworth Scale (MAS), and a strength rating was obtained with the Manual Muscle Test (MMT). The GMFM and Children Health Assessment Questionnaire (CHAQ) were used to assess gross motor function and HRQOL, respectively.

Spasticity was measured in the flexors and extensors of shoulder, elbow, along with the wrists of both the upper extremities. In both the lower extremities, spasticity was measured in the hip flexors and extensors, knee extensors, and ankle plantar flexors and dorsiflexors. The test was performed at moderate speed (180°/s) using standardized procedures. Intraclass correlation coefficients between 0.61-0.87 have been reported in children with spastic CP [27].

Strength was measured in the flexors and extensors of shoulder, elbow, and wrists of both the upper extremities, and in the hip flexors and extensors, knee extensors, and ankle plantar flexors and dorsiflexors of both the lower extremities. Reliability values of the MMT between 0.60-0.91 have been reported in children with hemiplegic CP [28].

Spasticity and strength were quantified by summating all MAS and MMT scores, respectively. MAS scores for all lower and upper extremities measurements were summated to provide a spasticity score. Likewise, MMT scores for all measurements were summated to provide a strength score. Both scores indicate the extent or magnitude of impairment.

GMFM, a standard criterion-referenced test designed to assess changes in gross motor function in children with CP, was used in this study [29]. The total number of items is 88, with five dimensions: lying and rolling, sitting, crawling and kneeling, standing, and walking, running, and jumping. A 4-point Likert scale is used for scoring each item. Acceptable reliability of the GMFM scores has been reported for children with CP [30], and Cronbach’s alpha coefficient was 0.960 in this study.

The HRQOL of school-aged children with CP was measured using the CHAQ. Physical therapists used the Korean version of the CHAQ to interview the parents. The CHAQ consists of eight domains: dressing/grooming, arising, eating, walking, hygiene, reach, grip, and activities. Questions are answered on a 4-point Likert scale from 0 to 3, where 0 means able to do with no difficulty, 1 able to do with some difficulty, 2 able to do with much difficulty, and 3 unable to do. The mean score of the 8 domains determines the CHAQ score. Cronbach’s alpha values ranging from 0.76 to 0.97 have been reported for the eight domains [31], with values of 0.880-0.973 in children with CP [32]. The CHAQ was administered by six physical therapists, each with at least 3 years’ experience in providing therapy for children with CP. The Korean version of the CHAQ was validated by Park [32] in a study with 72 children with CP. The Korean version of the CHAQ was translated according to standard translation procedures and reported psychometric properties including reliability, the floor and ceiling effect, and correlation with gross motor function measure [32].

**Fig. 1:**
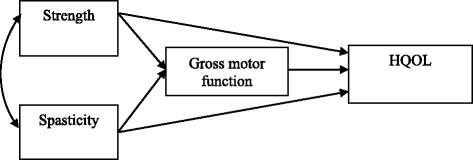
A recursive model. The variables in recursive model are shown. The arrow indicates the direction of the relationship between the variables

Figure 1 shows a recursive model to investigate the causal relationships between spasticity, strength, gross motor function, and HRQOL in school-aged children with spastic CP using path analysis. A recursive model is one with no reciprocal causations or feedback loops.

The variables included in the path model and the direction of the causal relationship were based on previous research and general logic. In this study, correlations of variables were analyzed before performing path analysis, which involved verifying the significant coefficients in the recursive model, and subsequently investigating the proposed path model fit indices. In path analysis, a goodness of fit test is performed in order to confirm that the model shows the best possible fit.

After testing the appropriateness of the model, direct, indirect, and total effects were examined through the bootstrap method. The AMOS 22.0 statistical program was employed to analyze the path models, obtain maximum-likelihood estimates of model parameters, and provide goodness-of-fit indices. The level of significance was set at 0.05.

## Results

Pearson correlation coefficients for all variables are presented Table 1. Four variables showed significant correlations (*p* < 0.001) in the expected directions. There was no multi-collinearity because bivariate correlations did not exceed 0.80 [33].

**Table 1:** Correlations among variables

	Strength	Spasticity	Gross motor function	CHAQ
Strength	–			
Spasticity	−.434**	–		
Gross Motor Function	.673**	−.654**	–	
HRQOL	−.677^**^	.488**	−.762**	–

**p < 0.01; HRQOL: Health Related Quality of Life

**Fig. 2:**
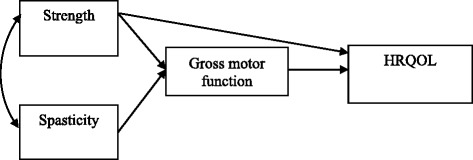
A proposed path model. Non-significant path coefficient is deleted in recursive model. The arrow indicates the direction of the relationship between the variables

To verify the model, preliminary path analyses were performed. The path coefficient of spasticity (β = − 0.084) to HRQOL was not significant (*p* > 0.05) (Table 2). Figure 2 shows the proposed path model.

**Table 2:** Path coefficients of the recursive model

Path	B	β	S.E.	C.R.
Gross motor function ← Strength	1.166	0.479	0.215	5.426**
Gross motor function ← Spasticity	−1.779	−0.446	0.352	−5.049**
HRQOL ← Gross motor function	−0.239	−0.616	0.048	−4.941**
HRQOL ← Strength	−0.283	−0.299	0.099	−2.861**
HRQOL ← Spasticity	−0.131	−0.084	0.159	−0.823

**p < .01

Multiple criteria were used for the evaluation of model fit. Fit indices include the theoretical meaningfulness of the model, absolute and incremental fit indices, and model cross-validation. Absolute fit indices are used to determine how well a model fits the data without comparing it to a baseline model, whereas incremental fit indices are used to investigate how much better the model fits than a baseline model. In this study, chi-square (χ^2^) and the root mean square error of approximation (RMSEA) were used as absolute fit indices, and the normed fit index (NFI) and the comparative fit index (CFI) as incremental fit indices. χ^2^ was not recommended as a judgment of model fit because it is sensitive to the sample size used in the analysis of model fit; therefore, it was only reported but not used as a fit statistic in this study.

As shown in Table 3, the proposed model showed excellent fit indices. RMSEA was lower than 0.05 and NFI and CFI were than greater 0.9, which indicates a good model fit.

**Table 3:** Model fit indices

Fit index	*χ* ^2^	RMSEA^a^	NFI^b^	CFI^c^
	.674	.000	.995	1.000

^a^Root Mean Square Error of Approximation

^b^Normed Fit Index

^c^Comparative Fit Index

The effect of spasticity, strength, and gross motor function on HRQOL in children with spastic CP was tested through path analysis (Table 4).

**Table 4:** Path coefficients of the proposed model

Path	B	β	S.E.	C.R.
Gross motor function ← Strength	1.166	.479	0.215	5.426**
Gross motor function ← Spasticity	−1.779	−.446	0.352	−5.049**
HRQOL ← Gross motor function	−0.218	−.560	0.041	−5.322**
HRQOL ← Strength	1.166	−.300	0.215	5.426**

**p < .01

HRQOL measured with CHAQ was the dependent variable. Exogenous independent variables were spasticity and strength. The endogenous independent variable as a mediator was gross motor function. As shown in Table 5, the direct effect of spasticity on gross motor function (−.446), of strength on gross motor function (.479), of strength on HRQOL (−.300), and of gross motor function on HRQOL (−.560) was statistically significant (*p* < 0.05). The indirect effect of spasticity on HRQOL (.250) and of strength on HRQOL (−.269) were also statistically significant (p < 0.05).

**Table 5:** Direct and indirect effects of variables

Predictor variables	Independent variables	Total effect	Direct effect	Indirect effect	*R* ^*2*^
Spasticity	Gross motor function	-..446*	-..446*		.614
Strength		.479*	.479*		
Spasticity	HRQOL	.250*		.250*	.630
Strength		−.569*	−.300*	−.269*	
Gross motor function		−.560*	−.560*		

*p < .05

## Discussion

Information about HRQOL might be useful to evaluate the effect of interventions on overall well-being [34]. Measures of HRQOL may be used to predict the future status of individuals with an illness or condition, and could thus be used as better indicators than functional outcome assessments in children with CP who attend school [35].

QOL is concerned with the most important aspects resulting from disability in terms of an individual’s level of independence in life roles. Almost all therapy is provided on the assumption that a reduction in motor impairment will result in an increase in everyday functioning and participation for children with CP. However, the causal relationship between motor impairment and functional outcome was seldom empirically demonstrated [36, 37], especially in relation to HRQOL. While factors contributing to functional outcomes, such as strength, spasticity, and gross motor function, are important to improve functional abilities in children with CP, there are insufficient studies on comprehensive relationship between these variables.

Some studies have identified fragmentary relationship between variables. Motor impairments such as spasticity, weakness, restricted range of motion, and selective motor control ability have an effect on functional ability in terms of gross motor function and daily activities outcomes [37–39]. A strong relationship between gross motor function and QOL has also been reported [16, 40]. However, no path analysis has been conducted to predict HRQOL in children with CP.

This study employed path analysis to test the causal relationship between spasticity, strength, gross motor function, and HRQOL in school-aged children with spastic CP. Path analysis is superior to regression analysis as it provides an explanation for both the causal relationship and the relative importance of alternative paths of influence [41]. The proposed model in this study aimed to determine the contribution of spasticity, strength, and gross motor function to HRQOL, and was revealed to have the best fit for these variables.

The present data confirm and extend previous findings indicating that spasticity and weakness are causal factors of gross motor function. Furthermore, gross motor function was shown to be a causal factor of HRQOL. The path coefficients of spasticity and strength to gross motor function were significant (*p* < 0.05). If the spasticity score is increased by 1 SD, total gross motor function score is reduced by 0.4 SD. These results are supported by previous studies that found a significant correlation between spasticity and gross motor function [39, 42, 43]. Moreover, if the strength score is increased by 1 SD, total gross motor function score is increased by 0.5 SD. This is consistent with previous studies in which muscle strength was found to be associated with motor function [37, 44]. An increase of 1 SD in gross motor function and strength scores produces a decrease of 0.560 and 0.3 SD in the CHAQ score, respectively. Because higher CHAQ scores (measuring HRQOL) indicate lower levels of HRQOL, the direction was the opposite of that of GMFM and strength scores.

Spasticity and strength were exogenous variables of gross motor function and accounted for substantial proportions of the variance in gross motor function (61.4%). Spasticity, strength, and gross motor function were explanatory variables of HRQOL and accounted for substantial proportions of the variance in CHAQ scores (63.0%). Although a percentage above 60 accounted for is large, it also suggests that other variables should be included in the model to explain over 30% of the variance in HRQOL. Direct effects of spasticity and strength on gross motor function, and strength and gross motor function on HRQOL were detected. A significant positive indirect effect of spasticity and a significant negative indirect effect of strength on HRQOL were also identified. Indirect effects include the subsequent effects of spasticity and strength as exogenous variables through another variable, i.e., gross motor function, on HRQOL, the endogenous variable. This is known as a mediating effect of gross motor function, as the proposed path model shows that spasticity and strength influence HRQOL through their effect on gross motor function.

This study provides primary evidence for the mediating role of gross motor function in the relationship of spasticity and strength with HRQOL. Spasticity and strength accounted for 61.4% of the variance in gross motor function; and spasticity, strength, and gross motor function accounted for 63.0% of the variance in HRQOL. The proportion of the variance in HRQOL explained by these variables included direct and indirect effects, as well as the mediating effect of gross motor function in relation of spasticity and strength with HRQOL. Furthermore, spasticity was completely mediated by gross motor function. The results of this study provide a starting point to establish causal relationships between motor impairment, gross motor function, and HRQOL. Although the results from path analysis in this study provide important insights into the links between spasticity, strength, gross motor function, and HRQOL, further research is needed to understand the nature of these relationships in more detail considering the ICF model.

There are limitations to this study. Firstly, the sample size is small; thus, further research needs to include more children with CP to increase the statistical power and clinical meaning of the present study findings. A second limitation is related to the age range of the study sample. Although this was limited to school age, it is possible that 9 years of motor development make an important contribution to HRQOL changes; therefore, a further study should be conducted controlling for age. Finally, parent interviews were conducted to assess children’s HRQOL in the present study. Although QOL must be measured by directly assessing the concerned individuals whenever possible, in cases of severe cognitive impairment, intellectual problems, or communication difficulties, parent-reported QOL is used to obtain reliable information about children’s QOL [21, 45]. In previous studies, the degree of parental stress was shown to be a factor affecting children’s HRQOL, but this study did not consider the role of parental factors, such as stress. In future studies, it is thus necessary to study the HRQOL of children with CP considering parental status.

## Conclusion

This study investigated the relationship between motor impairment, including strength and spasticity, gross motor function, and HRQOL using path analysis. Data were collected from 62 school-aged children with spastic CP. Significant paths from spasticity and strength to gross motor function and from strength and gross motor function to HRQOL were identified, and the role of gross motor function as a mediator was confirmed through path analysis in school-aged children with CP. This study provides initial evidence for the causal relationships between motor impairment, gross motor function, and HRQOL in children with spastic CP. In addition, the results of this study are meaningful in that the QOL is the final goal of health; the concept of ICF is assumed to be improved, as the limit of activity and the restriction of participation decreased in children with CP.

The author declares no conflict of interests. This research received no specific grant from any funding agency in the public, commercial, or not-for-profit sectors.

## Abbreviations

CFI: Comparative fit index
CHAQ: Childhood Health Assessment Questionnaire
CP: Cerebral palsy
GMFM: Gross Motor Function Measure
HRQOL: Health-related quality of life
ICF: International Classification of Functioning, Disability and Health
MAS: Modified Ashworth Scale
MMT: Manual Muscle Test
NFI: Normed fit index
QOL: Quality of life
RMSEA: Root mean square error of approximation

## References

[1] Robertson CM, Ricci MF, O’Grady K (2017). Prevalence estimate of cerebral palsy in northern Alberta: births, 2008-2010. Can J Neurol Sci.

[2] Smithers-Sheedy H, McIntyre S, Gibson C (2016). A special supplement: findings from the Australian cerebral palsy register, birth years 1993 to 2006. Dev Med Child Neurol.

[3] Sellier E, Platt MJ, Andersen GL (2016). Decreasing prevalence in cerebral palsy: a multi-site European population-based study, 1980 to 2003. Dev Med Child Neurol.

[4] Van Naarden Braun K, Doernberg N, Schieve L (2016). Birth prevalence of cerebral palsy: a population-based study. Pediatrics.

[5] Beckung E, Hagberg G (2002). Neuroimpairments, activity limitations, and participation restrictions in children with cerebral palsy. Dev Med Child Neurol.

[6] Rosenbaum P, Paneth N, Leviton A (2007). A report: the definition and classification of cerebral palsy April 2006. Dev Med Child Neurol.

[7] Bjornson K, McLaughlin J (2001). The measurement of health-related quality of life (HRQL) in children with cerebral palsy. Eur J Neurol.

[8] Butler C, Darrah J (2001). Effects of neurodevelopmental treatment (NDT) for cerebral palsy: an AACPDM evidence report. Dev Med Child Neurol.

[9] Stucki G, Cieza A, Ewert T (2002). Application of the international classification of functioning, disability and health (ICF) in clinical practice. Disabil Rehabil.

[10] Kim WH, Park EY (2011). Causal relation between spasticity, strength, gross motor function, and functional outcome in children with cerebral palsy: a path analysis. Dev Med Child Neurol.

[11] Ko IH, Kim JH, Lee BH (2013). Relationships between lower limb muscle architecture and activities and participation of children with cerebral palsy. J Exerc Rehabil.

[12] Vargus-Adams J (2005). Health-related quality of life in childhood cerebral palsy. Arch Phys Med Rehabil.

[13] Ko J, Lee B-H, Kim M (2011). Relationship between function and health-related quality of life of school-aged children with cerebral palsy. J Phys Ther Sci.

[14] WHOQOL Group (1995). The World Health Organization quality of life assessment (WHOQOL): position paper from the World Health Organization. So Sci Med.

[15] Guyatt GH, Feeny DH, Patrick DL (1993). Measuring health-related quality of life. Ann Intern Med.

[16] Majnemer A, Shevell M, Rosenbaum P (2007). Determinants of life quality in school-age children with cerebral palsy. J Pediatr.

[17] Houlihan CM, O'Donnell M, Conaway M (2004). Bodily pain and health-related quality of life in children with cerebral palsy. Dev Med Child Neurol.

[18] Findlay B, Switzer L, Narayanan U (2016). Investigating the impact of pain, age, gross motor function classification system, and sex on health-related quality of life in children with cerebral palsy. Dev Med Child Neurol.

[19] Arnaud C, White-Koning M, Michelsen SI (2008). Parent-reported quality of life of children with cerebral palsy in Europe. Pediatr.

[20] Tuzun EH, Guven DK, Eker L (2010). Pain prevalence and its impact on the quality of life in a sample of Turkish children with cerebral palsy. Disabil Rehabil.

[21] Dickinson HO, Parkinson KN, Ravens-Sieberer U (2007). Self-reported quality of life of 8-12-year-old children with cerebral palsy: a cross-sectional European study. Lancet.

[22] Machado CSM, Ruperto N, Silva CHM (2001). The Brazilian version of the childhood health assessment questionnaire (CHAQ) and the child health questionnaire (CHQ). Clin Exp Rheumatol.

[23] Ruperto N, Malattia C, Bartoli M (2004). Functional ability and physical and psychosocial well-being of hypermobile schoolchildren. Clin Exp Rheumatol.

[24] Selvaag AM, Lien G, SØrskaar D (2005). Early disease course and predictors of disability in juvenile rheumatoid arthritis and juvenile spondyloarthropathy: a 3 year prospective study. J Rheumatol.

[25] Brunner HI, Maker D, Grundland B, et al.: Preference-based measurement of health-related quality of life (HRQL) in children with chronic musculoskeletal disorders (MSKDs). Med Decis Mak, 2003, 23(4): 314-322. 10.1177/0272989X03256008 12926581

[26] Gall JP, Gall MD, Borg WR. Applying educational research: a practical guide. NY: Longman publishing. Group. 1999.

[27] Mutlu A, Livanelioglu A, Gunel MK (2008). Reliability of Ashworth and modified Ashworth scales in children with spastic cerebral palsy. BMC Musculoskelet Disord.

[28] Klingels K, De Cock P, Molenaers G (2010). Upper limb motor and sensory impairments in children with hemiplegic cerebral palsy: can they be measured reliably?. Disabil Rehabil.

[29] Russel D, Rosenbaum P, Gowland C (1993). Gross motor function measure manual. Hamilton.

[30] Nordmark E, Hägglund G, Jarnlo G (1997). Reliability of the gross motor function measure in cerebral palsy. Scand J Rehabil Med.

[31] Wulffraat N, Van der Net J, Ruperto N (2001). The Dutch version of the childhood health assessment questionnaire (CHAQ) and the child health questionnaire (CHQ). Clin Exp Rheumatol.

[32] Park EY (2010). Exploration of utility for Korean translation of the childhood health assessment questionnaire in children with cerebral palsy. J Child Spec Need.

[33] Kline RB (2015). Principles and practice of structural equation modeling.

[34] Guyatt GH, Naylor CD, Juniper E (1997). Users’ guides to the medical literature: XII. How to use articles about health-related quality of life. JAMA.

[35] Saigal S, Feeny D, Rosenbaum P (1996). Self-perceived health status and health-related quality of life of extremely low-birth-weight infants at adolescence. JAMA.

[36] Mayston MJ (2001). People with cerebral palsy: effects of and perspectives for therapy. Neural Plast.

[37] Ross SA, Engsberg JR (2007). Relationships between spasticity, strength, gait, and the GMFM-66 in persons with spastic diplegia cerebral palsy. Arch Phys Med Rehabil.

[38] Ohata K, Tsuboyama T, Haruta T (2008). Relation between muscle thickness, spasticity, and activity limitations in children and adolescents with cerebral palsy. Dev Med Child Neurol.

[39] Østensjø S, Carlberg EB, Vøllestad NK (2004). Motor impairments in young children with cerebral palsy: relationship to gross motor function and everyday activities. Dev Med Child Neurol.

[40] McCarthy ML, Silberstein CE, Atkins EA (2002). Comparing reliability and validity of pediatric instruments for measuring health and well-being of children with spastic cerebral palsy. Dev Med Child Neurol.

[41] Olobatuyi ME (2006). A user's guide to path analysis.

[42] Tuzson AE, Granata KP, Abel MF (2003). Spastic velocity threshold constrains functional performance in cerebral palsy. Arch Phys Med Rehabil.

[43] Damiano DL (2006). Activity, activity, activity: rethinking our physical therapy approach to cerebral palsy. Phys Ther.

[44] Verschuren O, Ketelaar M, Gorter JW (2009). Relation between physical fitness and gross motor capacity in children and adolescents with cerebral palsy. Dev Med Child Neurol.

[45] Gates P, Otsuka N, Sanders J (2010). Functioning and health-related quality of life of adolescents with cerebral palsy: self versus parent perspectives. Dev Med Child Neurol.

